# A Rapid ATP Bioluminescence-based Test for Detecting Levofloxacin Resistance Starting from Positive Blood Culture Bottles

**DOI:** 10.1038/s41598-019-49358-9

**Published:** 2019-10-02

**Authors:** Atsushi Matsui, Hideki Niimi, Yuichi Uchiho, Shunsuke Kawabe, Hideyuki Noda, Isao Kitajima

**Affiliations:** 1 First Department of Internal Medicine, Toyama University Hospital, Toyama, 930-0194 Japan; 2 Graduate School of Medicine and Pharmaceutical Sciences (medicine), University of Toyama, Toyama, 930-0194 Japan; 3 Hitachi, Ltd. Research & Development Group, Tokyo, 185-8601 Japan

**Keywords:** Bacterial infection, Laboratory techniques and procedures

## Abstract

Administering appropriate antimicrobial therapy as early as possible is important for rescuing bacteremic patients. Therefore, rapid antimicrobial susceptibility tests in positive blood culture specimens have been diligently sought. Adenosine triphosphate (ATP) bioluminescence-based methods have been used for rapid antimicrobial susceptibility tests. However, blood culture specimens have not been examined in many studies, possibly due to abundant intracellular ATP in blood corpuscles resulting in false-susceptible results. In this study, we developed a rapid ATP bioluminescence-based method for detecting antibiotic resistance starting from positive blood culture. To minimize background ATP originating from blood corpuscles, specimens were centrifuged and the supernatant diluted with broth, and an ATP-eliminating reagent was then added to the bacterial suspension at the beginning of incubation. This newly devised procedure reduced the background ATP by more than five orders of magnitude. In a pilot study using levofloxacin, no false-susceptible results were observed in 15 clinical specimens. Furthermore, the results indicated that the rapid method provided additional information about bacterial activities with high resolution, in contrast to the less-thorough findings with the conventional turbidity method. Therefore, our approach will contribute to the treatment of infectious diseases as a rapid antimicrobial susceptibility test.

## Introduction

Bacterial infections remain a medical burden, and despite the development of many classes of antibiotics, the burden has only increased with time, as the emergence of antibiotic resistant bacteria has become a worldwide problem. To avoid the further emergence of such bacteria and prevent treatment failure, it is important to administer appropriate antibiotics as early as possible in accordance with the results of microbiological tests. Therefore, a great deal of effort has been made to accelerate the return of results of microbiological tests. Some rapid identification methods for pathogenic bacteria have been established already. In particular, identification with matrix-assisted laser desorption ionization-time of flight mass spectrometry (MALDI-TOF MS) technology has become popular recently^1–4^. In addition, new rapid identification systems involving genetic techniques, such as the melting temperature mapping method, have also been developed^5^.

However, few rapid antimicrobial susceptibility tests have been established. MALDI-TOF MS and genetic techniques are useful for assessing antimicrobial susceptibility, but these methods cannot be applied to bacteria with resistance mechanisms induced by unknown proteins or genes^6–11^. The broth micro-dilution method and disk diffusion method are based on growth pattern and can be applied to such bacteria. Although the procedures recommended by the Clinical & Laboratory Standards Institute are global gold-standard antimicrobial susceptibility tests, they require isolates with overnight culture. Therefore, it can take two to three days after blood collection to obtain results. The consequent use of inappropriate antimicrobial agents in the initial treatment period often results in life-threatening conditions in patients with severe infections.

To overcome the problems mentioned above, several rapid antimicrobial susceptibility tests based on growth patterns have been developed. Accelerate Pheno system^12–15^ and several methods^16,17^ can rapidly determine antimicrobial susceptibility by detecting of bacterial growth using a microscopic imaging analysis. Several studies have reported that direct inoculation from positive blood culture bottles can be applied to the VITEK-2 and Phoenix system^18–21^. The procedure proposed by the European Committee on Antimicrobial Susceptibility Testing (EUCAST) has recently attracted attention as a rapid disk diffusion method, and its adapted versions are gradually being adopted.

On the other hand, some studies have focused on adenosine triphosphate (ATP) bioluminescence^22–25^. ATP is a common energy transfer compound in all organisms. Regarding bacterial suspension, a strong positive correlation has been observed between the bacterial count and the ATP level measured by bioluminescence^26^. Furthermore, measuring the ATP level would enable the more quantitative detection of bacterial growth than conventional turbidity methods. Previous reports have suggested that rapid antimicrobial susceptibility tests involving ATP bioluminescence are promising. Indeed, the tests have been applied to not only general bacteria but also other species, including acid-fast bacteria^27,28^, mycoplasma species^29^ and fungi^30^, with various classes of antibiotics^22,23,25,31^. However, only isolated bacteria or those in urine specimens were evaluated in those previous ATP bioluminescence studies. Although systemic bacterial infections, such as sepsis, are frequently accompanied by bacteremia and a more rapid antimicrobial susceptibility test would be preferable in such cases, specimens taken directly from positive blood culture bottles have not been examined yet. This is because the abundant intracellular ATP derived from blood corpuscles could become a great obstacle for detecting bacterial growth. In the present study, employing newly devised sample preparation procedures, we developed a rapid ATP bioluminescence-based method for detecting antibiotic resistance starting from positive blood culture bottles, and demonstrated rapid levofloxacin resistance detection.

## Results

### Workflow of the rapid ATP bioluminescence-based method for detecting levofloxacin resistance starting from positive blood culture bottles

**Figure 1:**
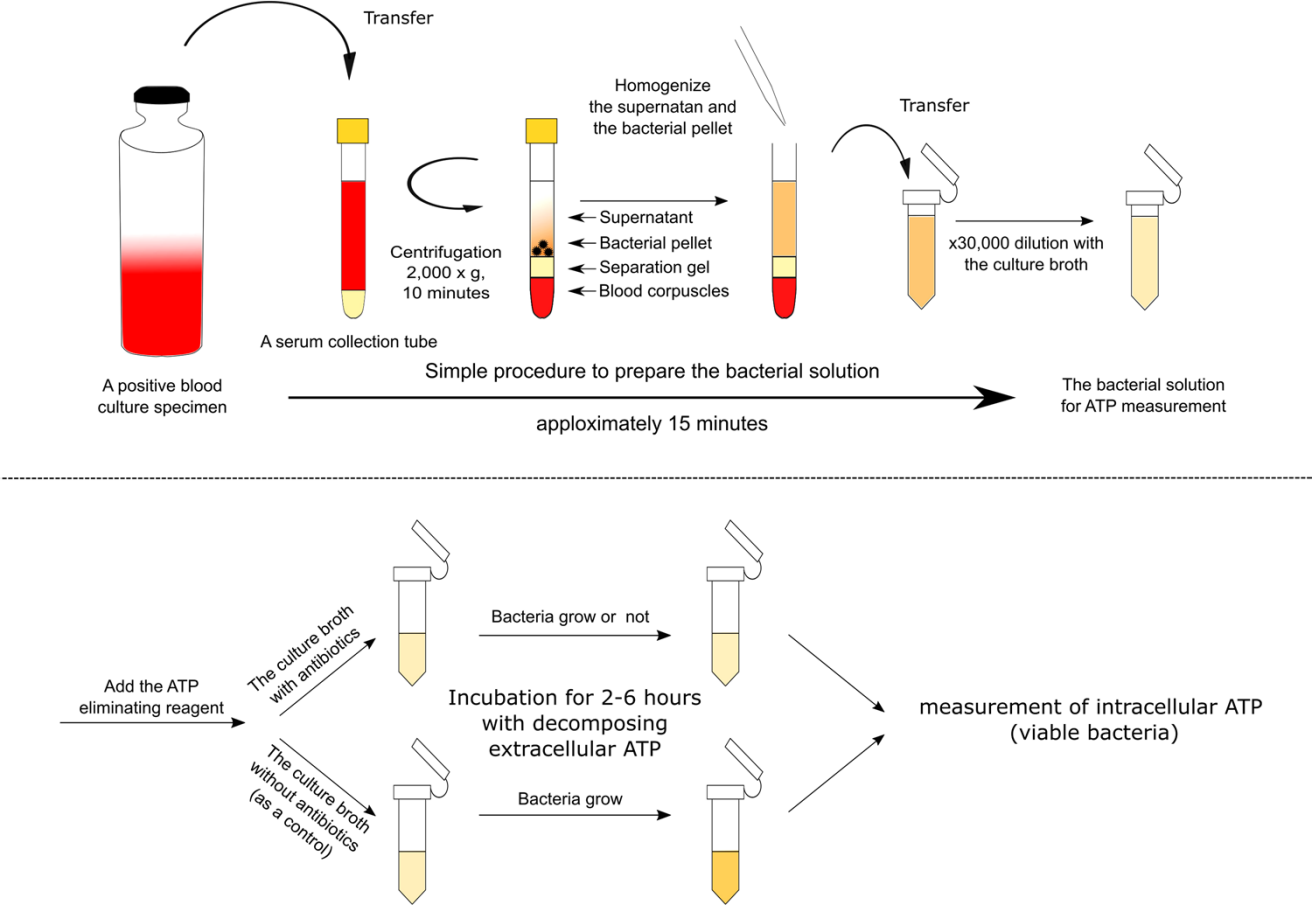
Simple procedure for preparing the bacterial solution for adenosine triphosphate (ATP) measurement.

The rapid ATP bioluminescence-based method consists of four steps (Fig. 1). First, to exclude blood corpuscles, a positive blood culture specimen in a serum separator tube is centrifuged, causing the erythrocytes and leukocytes to move under the separation gel of the tube. To obtain a bacterial solution for ATP measurement, the resulting supernatant and bacterial pellet are homogenized and diluted 30,000-fold with a culture broth. This first step takes no more than 15 minutes. Second, to reduce extracellular ATP in the broth, an ATP-eliminating reagent is added to the diluted suspension, and then the mixture is incubated with an antibiotic at various concentrations (or with the broth only as a control) for 2, 4 and 6 h. The ATP-eliminating reagent decomposes abundant extracellular ATP contained originally in the broth and the blood culture specimen or extracellular ATP excreted from growing bacteria while incubating. Third, intracellular ATP in bacteria is released with an ATP-releasing reagent after incubation, and the ATP-eliminating reagent is deactivated at the same time. Thus, a sample solution containing only intracellular ATP from growing bacteria is obtained. Finally, the ATP level of this solution is measured by ATP bioluminescence.

### The “background ATP”-reducing effect of the rapid method

“Background ATP” needs to be reduced as much as possible in order to detect small changes in bacterial intracellular ATP. In this context, “background ATP” refers to both the intracellular ATP contained in blood corpuscles and the ATP originally contained in culture broths. We therefore confirmed the background ATP-reducing effect of the rapid method with healthy blood culture specimens. With the simple procedure for preparing the bacterial solution for ATP measurement, the background ATP level contained in 10 µL of each sample solution was determined to be around 100 to 400 amol (Table 1). In contrast, without the simple process of removing blood corpuscles, the background ATP levels contained in the 10-µL sample solution derived from these specimens exceeded the measurement range (more than 10,000,000 amol). Thus, the values achieved with processing were roughly five orders of magnitude lower than those without processing.

**Table 1:** Background adenosine triphosphate (ATP): the ATP levels contained in 10 µL of sample solution derived from healthy culture-negative blood specimens.

Incubation time of blood culture	ATP levels (amol, mean ± standard deviation of triplicate processes)	
	Aerobic bottle	Anerobic bottle
6 h	256 ± 102	125 ± 34
24 h	289 ± 29	197 ± 51

Without removing blood corpuscles with centrifugation, the ATP background exceeded 10,000,000 amol.

### Levofloxacin resistance detection using clinical specimens

**Figure 2:**
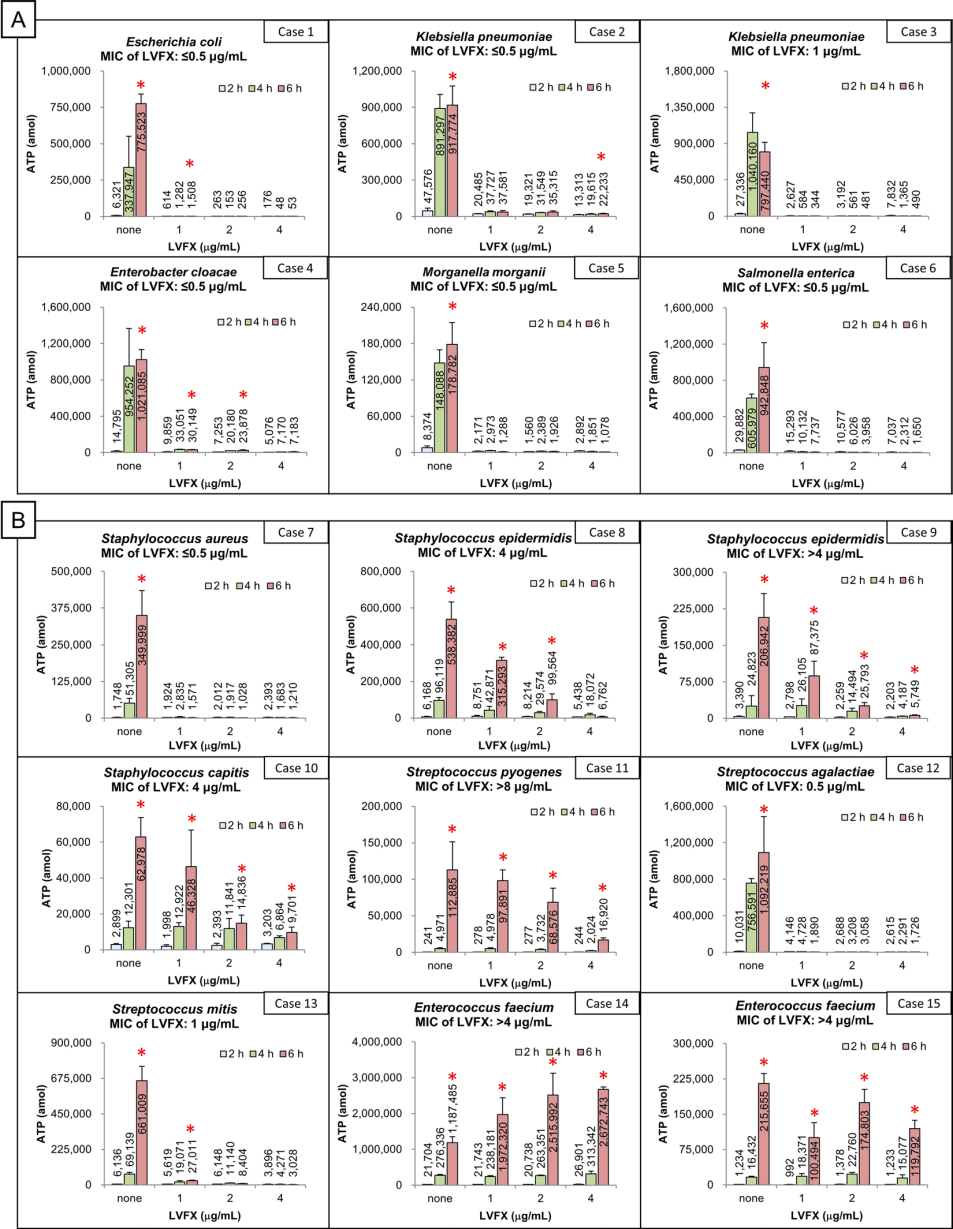
The results of clinical specimens obtained by the rapid adenosine triphosphate method. (**A**) Gram-negative strains. (**B**) Gram-positive strains. The ATP levels are shown as the means ± standard deviations of three independent extraction processes. Red asterisks indicate that a significant increase in the ATP level was observed at the six-hour time point with LVFX compared to two-hour time point with the same concentration of LVFX. MIC = minimal inhibitory concentration, LVFX = levofloxacin, ATP = adenosine triphosphate.

In this study, 15 clinical strains (15 cases) were tested starting directly from positive blood culture bottles. Figure 2 shows the changes in the ATP level contained in 10 µL of sample solution after incubation with levofloxacin (LVFX), or without LVFX (as a control) by the clinical strain (refer to the Supplementary Table S1, described in more detail). Red asterisks indicate that a significant increase in ATP was observed at the 6-h time point with LVFX compared to 2-h time point with the same concentration of LVFX. For example, in case 8, the ATP level did not increase with 4 µg/mL of LVFX, whereas the levels significantly increased with 1 and 2 µg/mL of LVFX at the 6-h time point. Therefore, the minimal inhibitory concentration (MIC) value obtained by our method was confirmed to be 4 µg/mL, which was the same as the value obtained by the conventional broth microdilution method. The MIC values obtained by the rapid method were consistent with those of the conventional method in 10 of 15 cases (Table 2). In the other 5 cases (cases 1, 2, 4, 10 and 13), the MIC values obtained by the rapid method were higher than those by the conventional method. However, although the ATP level significantly increased with 4 µg/mL of LVFX in case 2, it did not increase with 1 or 2 µg/mL of LVFX. Therefore, the so-called skip phenomenon was observed in case 2.

**Table 2:** The minimal inhibitory concentration values of clinical blood culture specimens obtained by the rapid ATP method and the conventional method.

Cases#	Species	MIC values of LVFX (μg/mL)		consistency
		Rapid ATP method^*a^	Conventional method^*b^	
1	*Escherichia coli*	2	≤0.5	more resistance
2	*Klebsiella pneumoniae*	>4	≤0.5	more resistance^*c^
3	*Klebsiella pneumoniae*	≤1	1	consistent
4	*Enterobacter cloacae*	4	≤0.5	more resistance
5	*Morganella morganii*	≤1	≤0.5	consistent
6	*Salmonella enterica*	≤1	≤0.5	consistent
7	*Staphylococcus aureus*	≤1	≤0.5	consistent
8	*Staphylococcus epidermidis*	4	4	consistent
9	*Staphylococcus epidermidis*	>4	>4	consistent
10	*Staphylococcus capitis*	>4	4	more resistance
11	*Streptococcus pyogenes*	>4	>8	consistent
12	*Streptococcus agalactiae*	≤1	0.5	consistent
13	*Streptococcus mitis*	2	1	more resistance
14	*Enterococcus faecium*	>4	>4	consistent
15	*Enterococcus faecium*	>4	>4	consistent

^*a^Performed with 1–4 µg/mL of LVFX. ^*b^Performed with 0.5–4 µg/mL of LVFX for all species except *Streptococci*. For *Streptococci*, 8 µg/mL was also tested. ^*c^Bacterial growth was observed only with 4 µg/mL of LVFX (skip phenomenon).

MIC = minimal inhibitory concentration, LVFX = levofloxacin, ATP = adenosine triphosphate.

### Levofloxacin resistance detection using artificially positive blood culture specimens from clinical isolates

Ten of the 15 strains of clinical specimens were highly resistant or susceptible. The MIC values of such strains can be determined by any method. Therefore, we performed additional experiments with artificial positive blood culture specimens from 8 clinical isolates of Enterobacterales near the breakpoint of susceptible/resistant (1–4 µg/mL for LVFX). The results are shown in Table 3 (refer to Supplementary Table S2, described in more detail). In 5 strains, the MIC values were consistent with those of the conventional method, whereas the MIC values were higher than those of the conventional method in 3 strains (strains 2, 3 and 6).

**Table 3:** The minimal inhibitory concentration values of clinical isolates obtained by the rapid ATP method and the conventional method.

Strains#	Species	MIC values of LVFX (μg/mL)*		consistency
		Rapid ATP method	Conventional method	
1	*Escherichia coli*	1	1	consistent
2	*Escherichia coli*	4	2	more resistance
3	*Escherichia coli*	>4	4	more resistance
4	*Klebsiella pneumoniae*	2	2	consistent
5	*Klebsiella pneumoniae*	2	2	consistent
6	*Klebsiella pneumoniae*	>4	4	more resistance
7	*Klebsiella oxytoca*	1	1	consistent
8	*Citrobacter freundii*	1	1	consistent

^*^Performed with 0.5–4 µg/mL of LVFX in both methods.

MIC = minimal inhibitory concentration, LVFX = levofloxacin, ATP = adenosine triphosphate.

## Discussion

To rescue bacteremic patients and prevent the emergence of further drug-resistant bacteria, it is vital to administer appropriate antibiotics as early as possible. Therefore, rapid antimicrobial susceptibility tests have long been sought. We considered that the rapid ATP bioluminescence-based method would be useful for developing novel rapid antimicrobial susceptibility tests starting directly from positive blood culture bottles.

The abundant intracellular ATP in blood corpuscles, such as erythrocytes and leukocytes, has been a large obstacle to performing a rapid ATP test. Although intracellular ATP in bacteria is released with an ATP-releasing reagent in standard ATP bioluminescence methods (as well as our own method), intracellular ATP in blood corpuscles is also released at the same time. Therefore, abundant intracellular ATP derived from blood corpuscles masks relatively small amounts of intracellular ATP from growing bacteria. We resolved this issue by incorporating the original process, wherein blood corpuscles were moved under the separation gel of the serum collection tube by centrifugation. This modification reduced the background ATP levels to a low range (around 100 to 400 amol), resulting in an improved signal-to-background ratio. As another modification, ATP-eliminating reagent was added to the diluted suspension before incubation in the rapid method. This reagent is added to each aliquot portion *after* incubation in the standard ATP bioluminescence method^25,32,33^. Therefore, according to the standard method, an additional reaction time (30 minutes) of the reagent is necessary at each time point after incubation, which may carry a risk of further experimental errors. On the other hand, with our method, the reagent in the suspension decomposes extracellular ATP during incubation, thus making it possible to avoid the additional reaction time. Furthermore, the sample preparation procedures can prevent such experimental errors and reduce the cost and hands-on time. Although we were not able to examine the effect of this reagent on growth of all species in this study, no obvious effect was observed in the combination of LVFX and 12 species at least.

The MIC values obtained by the rapid method were consistent with those obtained by the conventional method in 10 out of 15 cases. For the inconsistent 5 cases (case 1, 2, 4, 10, and 13), the MIC values were higher than that by the conventional method. For example, in case 4, the ATP levels significantly increased with 1 and 2 µg/mL of LVFX at the 6-h time point, although the MIC value was ≤0.5 ug/ml when using the conventional method. Thus, the values obtained by the rapid method tended to be higher than those of the conventional method overall. This tendency toward a higher resistance being detected was similarly observed in additional experiments using artificially positive blood culture specimens from clinical isolates.

The tendency can be attributed to two reasons. First, filamentation of bacteria is an SOS response against antibiotics, as was mentioned in previous reports^31,33,34^. Because filamentous cells do not divide under antibiotic treatment, they do not show sufficient turbidity to be detected with the conventional method. However, the ATP level contained in a filamentous cell is more than 20-fold that in a steady-state bacterium^33^. Since filamentous cells start to divide into their normal forms and grow again after antibiotics are removed or deactivated^32,33^, it may lead to treatment failure after discontinuation of antibiotics. Our method can therefore prevent treatment failure due to the filamentation of bacteria by detecting it as an increase in intracellular ATP. Second, the sensitivity differs between the two methods. Antimicrobial susceptibility tests by ATP bioluminescence observe bacterial growth with a molecular biological approach, whereas tests by the conventional method observe it with a physicochemical approach; the former therefore observes bacterial growth more sensitively than the latter. We successfully reduced the background ATP levels with our procedure, which is why the rapid method was able to detect these subtle changes in ATP levels.

Our method may thus improve the treatment of bacteremia in certain situations. For example, in case 4, the ATP level increased more than 50-fold without LVFX. However, although the MIC value was 4 µg/mL, the level increased only approximately 3-fold with 1 and 2 µg/mL of LVFX. These findings indicate that bacterial growth is not completely inhibited by 2 µg/mL of LVFX alone. It is indeed rational to administer LVFX to immunocompetent hosts with bacteremia of this strain in accordance with the results of the conventional method. However, in severely immunocompromised hosts, such as transplant patients and hosts with severe combined immune deficiency, such an approach might lead to treatment failure. Detecting drug-resistant bacteria sensitively using our method may help prevent such treatment failure, even when due to host factors.

In the statistical analysis of this study, we compared the 6-h time point with the 2-h time point with the same concentration of LVFX. Comparing the 4-h time point with the 2-h time point with the same concentration of LVFX was also an option. In almost half of cases, the MIC values were consistent with those of the conventional method (refer to Supplementary Table S3). However, higher MIC values were observed than at the 6-h time point in other cases. Furthermore, in the control aliquot (without LVFX) of case 9, significant increase in ATP was not observed at the 4-h time point. This means that the bacteria did not grow significantly, and that the test itself was a failure at this time point. Therefore, we chose the 6-h time point as the point of judgement in this study. Although only 12 species were examined in this study, if experimental errors can be reduced through mechanization, judgement at an earlier time point than 6 h may be an option for certain species. The comparison of the 6-h time point with LVFX and the same time point without LVFX was also an option for the judgement algorithm. However, the lack of data concerning the cutoff point did not allow for the determination of whether or not bacteria grow, at this time. Further examinations would give us more information about judgement algorithms.

A large number of rapid antimicrobial susceptibility tests have been reported. Because antimicrobial susceptibility tests are performed in order to determine the most appropriate antibiotics, they should not be limited to antimicrobials if possible. Genetic techniques (GeneXpert System, Verigene System, etc.), immunochromatographic assays, and MALDI-TOF MS techniques are rapid, but these methods have limitations with respect to antibiotics and cannot be applied to bacteria with resistance mechanisms induced by unknown proteins or genes^6–11^. However, these limitations do not apply in theory to antimicrobial susceptibility test based on growth patterns. The EUCAST rapid antimicrobial susceptibility test, Accelerate Pheno system^12–15^, VITEK2 system^19–21^, and BD Phoenix system^18^ are such tests that have demonstrated rapidity. Although the VITEK2 system^19–21^ and BD Phoenix system^18^ were originally applied to isolates, methods using blood culture specimens directly have recently been reported. Good performance has been observed when using direct blood culture samples in both of those models, However, although in a few strains, “very major errors” were also observed^18–21^. Although we examined only the combination of LVFX and 12 species, “very major error” was not observed. Several studies have already reported that the ATP bioluminescence method can be applied to various classes of antibiotics or other species. Although further large-scale studies are needed, our approach may be able to provide a rapid antimicrobial susceptibility test with perfect sensitivity to the resistance described above.

Several limitations associated with the present study warrant mention. First, we did not examine polymicrobial specimens in order to simplify the evaluation of the rapid method. In polymicrobial specimens, the results of antimicrobial susceptibility testing would involve the summation of results of all of the kinds of bacteria in the blood of the bacteremia patient. In this case, a result of “susceptible” would mean that all kinds of bacteria in the blood are susceptible to the antibiotic. In contrast, a result of “resistant” would mean that at least one kind of bacteria in the blood was resistant to the antibiotic. Second, the bacterial counts in the suspension are irregular with our method, depending on the growth of bacteria in bottles. In this study where LVFX was examined, the concentration of inoculums did not have a remarkable influence on the MIC values. However, previous studies have reported that the concentration of inoculums affected the MIC values with the conventional method when evaluating combinations of beta-lactamase-producing strains and beta lactam-based antibacterials^35–37^. In our opinion, the concentration of inoculums affects the total production of beta-lactamases, which hydrolyze and directly decompose the beta lactam-based antibacterials. On the other hand, the main resistance mechanisms against LVFX are mutations of target proteins (Topoisomerase IV and DNA gyrase) and the activation of efflux pumps^38^. These mechanisms do not need protein synthesis, and therefore are not affected by the concentration of inoculums unlike in the case of beta lactamase. Third, the algorithm used to determine the MIC values was not thoroughly validated in this study. Validation study is necessary in the large-scale examination.

In conclusion, employing newly devised sample preparation procedures, we developed a rapid ATP bioluminescence-based method for detecting antibiotic resistance starting from positive blood culture bottles, and demonstrated rapid levofloxacin resistance detection. This procedure was completed within approximately six hours of the collection of positive blood culture specimens. Furthermore, the rapid method provided additional information about bacterial activities with high resolution, in contrast to the less-thorough findings with the conventional turbidity method. In the future, we are planning to conduct a large-scale examination with various classes of antibiotics (beta lactam-based antibacterials, macrolides, aminoglycosides, etc.) in order to examine the effect of the inoculum concentration on the MIC values and investigate the algorithm to determine the MIC values.

## Methods

### Ethics

All procedures were performed under a protocol approved by the Ethics Committee at the University of Toyama. Regarding residual specimens of a positive blood culture bottle, informed consent was obtained in the form of opt-out on Toyama University Hospital website from all participants. Regarding healthy blood, written informed consent was obtained from a healthy subject. The methods were carried out in accordance with the approved guidelines.

### Preparation of LVFX solution

High-performance liquid chromatography (HPLC)-grade LVFX powder (Sigma-Aldrich Japan, Tokyo, Japan) was dissolved and diluted with a cation-adjusted Mueller Hinton broth supplemented with 1% horse defibrinated blood (Nikken Bio., Kyoto, Japan) to obtain 10 µg/mL, 20 µg/mL or 40 µg/mL of LVFX solution.

### Confirmation of the background ATP-reducing effect

A 10 mL aliquot of healthy venous blood was added to both aerobic and anaerobic bottles. We used BacT/Alert^®^ FA (bioMerieux, Durham, NC, USA) as an aerobic bottle and BacT/Alert^®^ FN (bioMerieux) as an anaerobic bottle. These bottles were incubated for 6 or 24 h at 35 °C. After incubation, the healthy culture-negative specimens were processed with the same procedures as clinical specimens as described below, except for reaction time (for 1 h rather than 2 h) with the ATP-eliminating reagent.

### Clinical specimens

We collected 15 residual specimens of a positive blood culture bottle (BacT/Alert^®^ FA or BacT/Alert^®^ FN) from adult patients with bacteremia at Toyama University Hospital after the detection of bacterial growth with the BacT/ALERT 3D system (bioMerieux, Inc., Mercy-l’Etoile, France). All specimens were confirmed to be monomicrobial with Gram staining before use, because conventional antimicrobial susceptibility (and even identification) had not been completed at the time of enrollment. However, they were eventually confirmed to be monomicrobial using solid media at the Clinical Laboratory Center (certified ISO15189) at Toyama University Hospital. The MIC values determined by the conventional method, as reference values, were determined with the MicroScan WalkAway system (Siemens Healthcare Diagnostics, IL, USA) in the clinical laboratory.

### Preparation of a bacterial solution for ATP measurement

We injected 5 mL of a specimen from a culture bottle into a serum collection tube, BD Vacutainer SST™ II *Advance* (Nippon Becton Dickinson Co., Ltd., Tokyo, Japan). The tube was centrifuged at 2,000 × g for 10 minutes to remove the blood corpuscles under the separation gel. The resulting supernatant and bacterial pellet were homogenized and then diluted 30,000 times with the broth, to obtain a bacterial solution for ATP measurement.

### Eliminating extracellular free ATP and incubation

We added 100 µL of ATP-eliminating reagent solution attached to Lucifell HS Set (Kikkoman Biochemifa Co., Ltd., Tokyo, Japan) to 900 µL of the diluted bacterial solution. Four of the 180-µL aliquots of this mixture were incubated with 20 µL of the broth (as control) and the 3 concentrations of LVFX solution (the final levels were 1 µg/mL, 2 µg/mL and 4 µg/mL, respectively) at 35 °C.

### Release of intracellular ATP in bacteria after incubation

After 2-, 4- or 6-h incubation (or after only 1-h incubation, in the case of healthy blood culture specimens), 10 µL of each incubated bacterial solution was independently sampled 3 times. ATP-releasing reagent solution (10 µL) attached to Lucifell HS Set was added to each of the 3 solutions to obtain 20 µL of sample solution containing bacterial intracellular ATP, and then 10 of the 20 µL of each sample solution was loaded into a sample-socket of the measurement device described below in order to measure the ATP level.

### Measurement of the ATP levels

A fully automated and highly sensitive ATP measurement device “Lumione BL-2000” (Hitachi High-Tech Solutions Corporation, Tokyo, Japan) was used to measure the ATP levels. We selected the Luciferin-luciferase reagent HS attached to Lucifell HS Set, as a luminescent reagent, and diluted this reagent 50-fold with distilled water from the view point of experimental costs before use. We determined the ATP level contained in 10 µL of each sample solution using a standard curve. We prepared the curve from the volume of luminescence in the reference ATP solution included in the Lucifell ATP Standard Reagent Set (Kikkoman Biochemifa Co., Ltd., Tokyo, Japan) with the common logarithm of the volume of bioluminescence on the vertical axis and the common logarithm of ATP concentration on the horizontal axis. The absolute value of the correlation coefficient of the standard curve was greater than 0.999 in the range of 2.0 × 10^6^–2.0 × 10^11^ amol/L (equivalent to 20-2,000,000 amol in 10 µL).

### Definition of the MIC values in the rapid method

We defined the MIC value of each clinical strain as the minimum concentration of LVFX only if a significant increase in the ATP level at the six-hour time point was not observed.

### Preparation of artificial positive blood culture specimens from clinical isolates

We searched the database of clinical isolates stored in the clinical laboratory. Eight clinical isolates of Enterobacterales near the breakpoint of susceptible/resistant (1–4 µg/mL for LVFX) were available. These stored isolates were cultured using solid media to obtain colonies. Bacterial suspensions (0.5 McFarland) were adjusted from the colonies, and the suspensions were then diluted with the broth as appropriate. Subsequently, proximately 100 colony-forming units of bacteria were directly added to BacT/Alert® FA, and the bottles were incubated in the BacT/ALERT 3D system.

### Statistical analyses

The EZR software program, version 1.37 (Saitama Medical Center, Jichi Medical University) was used to process the experimental data. The ATP levels are presented as the means ± standard deviations of three sample solution that were independently sampled from each aliquot portion. The unpaired upper one-sided Welch’s *t*-test with a 0.05 significance level was used for the statistical analysis, with the ATP levels after six-hour incubation with various concentration of LVFX (and the broth as a control) compared to those after two-hour incubation with the broth.

## Supplementary information

Supplementary data

## Data Availability

The datasets generated during and/or analyzed during the current study are available from the corresponding author on reasonable request.
