# Supplementary material for: A Rapid ATP Bioluminescence-based Test for Detecting Levofloxacin Resistance Starting from Positive Blood Culture Bottles
Source: Sci Rep. 2019 Oct 2;9:13565. doi: 10.1038/s41598-019-49358-9 (PMC6775087; doi:10.1038/s41598-019-49358-9)
Supplement: Supplementary file 1 — Supplementary data [file 41598_2019_49358_MOESM1_ESM.pdf]

Supplementary Information

A Rapid ATP Bioluminescence-based Test for Detecting Levofloxacin Resistance Starting from Positive Blood Culture Bottles

Atsushi Matsui[1], Hideki Niimi[2*], Yuichi Uchiho[3], Shunsuke Kawabe[3], Hideyuki Noda[3], Isao Kitajima[2]

1. First Department of Internal Medicine, Toyama University Hospital, Toyama 930-0194, Japan

2. Graduate School of Medicine and Pharmaceutical Sciences (medicine), University of Toyama, Toyama 930-0194, Japan

3. Hitachi, Ltd. Research & Development Group, Tokyo 185-8601, Japan

*Correspondence to:

Hideki Niimi, M.D. Ph.D.

Graduate School of Medicine and Pharmaceutical Sciences (medicine), University of Toyama,

2630 Sugitani Toyama 930-0194 JAPAN

Phone: (+81)-76-434-7759, Fax: (+81)-76-434-7759

E-mail address: hiniimi@med.u-toyama.ac.jp

Supplemental Table S1. The detailed results of clinical specimens obtained by the rapid adenosine triphosphate method.

Cases #	Species (MIC values)	LVFX (μg/mL)	ATP levels (amol, mean ± standard deviation of triplicate processes) 2 hours			4 hours			6 hours			*p*-value
1	*Escherichia coli* (≤0.5 μg/mL)	4	176	±	116	48	±	13	53	±	13	0.90
		2	263	±	65	153	±	95	256	±	153	0.52
		1	614	±	103	1,282	±	242	1,508	±	102	<0.001
		none	6,321	±	357	337,947	±	212,270	775,523	±	66,139	0.001
2	*Klebsiella pneumoniae* (≤0.5 μg/mL)	4	13,313	±	2,409	19,615	±	4,408	22,233	±	3,336	0.011
		2	19,321	±	2,231	31,549	±	1,499	35,315	±	12,078	0.072
		1	20,485	±	2,874	37,727	±	9,045	37,581	±	12,142	0.064
		none	47,576	±	21,100	891,297	±	116,096	917,774	±	157,776	0.005
3	*Klebsiella pneumoniae* (1 μg/mL)	4	7,832	±	388	1,365	±	532	490	±	213	1.00
		2	3,192	±	393	561	±	262	481	±	214	0.99
		1	2,627	±	1,447	584	±	51	344	±	87	0.94
		none	27,336	±	6,571	1,040,160	±	240,079	1,018,628	±	116,663	0.004
4	*Enterobacter cloacae* (≤0.5 μg/mL)	4	5,076	±	793	7,170	±	280	7,183	±	2,317	0.13
		2	7,253	±	1,886	20,180	±	1,254	23,878	±	6,998	0.023
		1	9,859	±	889	33,051	±	3,522	30,149	±	3,967	0.005
		none	14,795	±	7,700	954,252	±	411,631	1,021,085	±	112,748	0.002
5	*Morganella morganii* (≤0.5 μg/mL)	4	2,892	±	303	1,851	±	554	1,078	±	304	1.00
		2	1,560	±	15	2,389	±	123	1,926	±	452	0.15
		1	2,171	±	296	2,973	±	608	1,288	±	450	0.97
		none	8,374	±	2,840	148,088	±	21,446	178,782	±	35,658	0.007
6	*Salmonella enterica* (≤0.5 μg/mL)	4	7,037	±	436	2,312	±	451	1,650	±	238	1.00
		2	10,577	±	2,159	6,026	±	1,307	3,958	±	1,837	0.99
		1	15,293	±	5,566	10,132	±	631	7,737	±	1,171	0.93
		none	29,882	±	3,423	605,979	±	42,635	942,848	±	273,767	0.014
7	*Staphylococcus aureus* (≤0.5 μg/mL)	4	2,393	±	848	1,683	±	848	1,210	±	1,337	0.86
		2	2,012	±	712	1,917	±	1,221	1,028	±	512	0.93
		1	1,924	±	123	2,835	±	2,073	1,571	±	588	0.80
		none	1,748	±	1,122	51,305	±	16,614	349,999	±	84,438	0.009

Cases #	Species (MIC values)	LVFX (μg/mL)	ATP levels (amol, mean ± standard deviation of triplicate processes)									*p*-value
			2 hours			4 hours			6 hours			
8	*Staphylococcus epidermidis* (4 μg/mL)	4	5,438	±	1,077	18,072	±	7,086	6,762	±	2,355	0.22
		2	8,214	±	2,004	29,574	±	5,817	99,564	±	32,200	0.019
		1	8,751	±	5,250	42,871	±	21,309	315,293	±	17,121	<0.001
		none	6,168	±	3,190	96,119	±	17,017	538,382	±	95,427	0.005
9	*Staphylococcus epidermidis* (>4 μg/mL)	4	2,203	±	434	4,187	±	163	5,749	±	1,192	0.012
		2	2,259	±	472	14,494	±	5,951	25,793	±	6,670	0.012
		1	2,798	±	256	26,105	±	13,424	87,375	±	30,288	0.020
		none	3,390	±	1,119	24,823	±	21,817	206,942	±	49,432	0.010
10	*Staphylococcus capitis* (4 μg/mL)	4	3,203	±	419	6,864	±	871	9,701	±	2,899	0.029
		2	2,393	±	1,248	11,841	±	5,613	14,836	±	4,479	0.016
		1	1,998	±	766	12,922	±	2,240	46,328	±	20,414	0.032
		none	2,899	±	492	12,301	±	3,593	62,978	±	10,830	0.005
11	*Streptococcus pyogenes* (>8 μg/mL)	4	244	±	31	2,024	±	298	16,920	±	2,523	0.004
		2	277	±	34	3,732	±	666	68,576	±	19,355	0.013
		1	278	±	7	4,978	±	1,158	97,891	±	15,052	0.004
		none	241	±	56	4,971	±	1,271	112,885	±	38,607	0.018
12	*Streptococcus agalactiae* (0.5 μg/mL)	4	2,615	±	357	2,291	±	409	1,726	±	218	0.99
		2	2,688	±	745	3,208	±	478	3,058	±	617	0.27
		1	4,146	±	602	4,728	±	88	1,890	±	1,173	0.97
		none	10,031	±	1,358	756,591	±	49,250	1,092,219	±	393,643	0.021
13	*Streptococcus mitis* (1 μg/mL)	4	3,896	±	1,145	4,271	±	579	3,028	±	264	0.84
		2	6,148	±	378	11,140	±	1,267	8,404	±	1,811	0.079
		1	5,619	±	355	19,071	±	7,168	27,011	±	4,320	0.006
		none	6,136	±	321	69,139	±	9,741	661,009	±	90,082	0.003
14	*Enterococcus faecium* (>4 μg/mL)	4	26,901	±	6,394	313,342	±	74,189	2,672,743	±	71,597	<0.001
		2	20,738	±	2,013	263,351	±	20,524	2,515,992	±	607,596	0.010
		1	21,743	±	4,480	238,181	±	24,065	1,972,320	±	465,704	0.009
		none	21,704	±	1,740	276,336	±	23,418	1,187,485	±	163,342	0.003
15	*Enterococcus faecium* (>4 μg/mL)	4	1,233	±	110	15,077	±	6,786	119,792	±	17,708	0.004
		2	1,378	±	581	22,760	±	4,050	174,803	±	27,762	0.004
		1	992	±	160	18,371	±	5,776	100,494	±	31,580	0.016
		none	1,234	±	264	16,432	±	2,331	215,655	±	20,832	0.002

The unpaired upper one-sided Welch's *t*-test with a 0.05 significance level was used for the statistical analysis, with the ATP levels after 6-h incubation with levofloxacin (or the broth as a control) compared to those after 2-h incubation with the same concentration of LVFX. MIC = minimal inhibitory concentration, LVFX = levofloxacin, ATP = adenosine triphosphate.

Supplemental Table S2. The detailed results of eight clinical isolates of Enterobacterales obtained by the rapid adenosine triphosphate method.

Isolates #	Species (MIC values)	LVFX (μg/mL)	ATP levels (amol, mean ± standard deviation of triplicate processes)			*p*-value
			2 hours	4 hours	6 hours	
1	*Escherichia coli* (1 μg/mL)	4	5,916 ± 1,149	2,078 ± 385	1,082 ± 192	0.992
		2	10,749 ± 2,591	5,379 ± 1,195	3,332 ± 191	0.981
		1	20,104 ± 7,423	20,876 ± 3,892	12,706 ± 1,959	0.111
		0.5	25,385 ± 9,458	1,330,235 ± 300,866	1,415,274 ± 98,913	0.001
		none	61,855 ± 14,205	1,573,583 ± 117,091	3,694,023 ± 233,487	0.001
2	*Escherichia coli* (2 μg/mL)	4	27,892 ± 6,931	14,563 ± 3,224	5,021 ± 1,120	0.99
		2	33,835 ± 7,017	549,680 ± 114,155	1,330,779 ± 281,906	0.008
		1	35,698 ± 8,416	1,041,079 ± 271,590	1,275,234 ± 242,381	0.006
		0.5	38,688 ± 5,238	1,195,430 ± 300,783	1,535,410 ± 265,404	0.005
		none	43,597 ± 1,042	1,234,942 ± 278,006	2,341,896 ± 506,182	0.008
3	*Escherichia coli* (4 μg/mL)	4	70,458 ± 13,361	217,457 ± 20,391	146,633 ± 18,579	0.003
		2	78,785 ± 12,347	1,441,618 ± 292,193	1,445,715 ± 61,718	<0.001
		1	74,173 ± 16,408	1,429,284 ± 321,717	1,442,828 ± 34,144	<0.001
		0.5	72,828 ± 13,225	1,468,581 ± 289,086	1,434,589 ± 52,411	<0.001
		none	91,928 ± 10,640	1,947,389 ± 321,862	1,849,431 ± 147,323	0.001
4	*Klebsiella pneumoniae* (2 μg/mL)	4	6,776 ± 1,941	826 ± 271	420 ± 425	0.99
		2	9,091 ± 2,537	2,383 ± 328	950 ± 381	0.99
		1	8,662 ± 2,640	132,262 ± 1,151	1,163,799 ± 538,042	0.033
		0.5	8,514 ± 2,424	251,545 ± 12,721	2,302,930 ± 446,472	0.006
		none	11,981 ± 2,748	324,908 ± 9,779	2,502,559 ± 469,825	0.006

Isolates #	Species (MIC values)	LVFX (μg/mL)	ATP levels (amol, mean ± standard deviation of triplicate processes)			p-value
			2 hours	4 hours	6 hours	
5	*Klebsiella pneumoniae* (2 μg/mL)	4	28,810 ± 1,216	7,821 ± 513	5,386 ± 121	1.00
		2	38,025 ± 1,361	40,826 ± 712	32,257 ± 341	0.99
		1	40,184 ± 704	404,727 ± 1,905	2,602,649 ± 93,190	<0.001
		0.5	41,077 ± 792	722,824 ± 48,230	2,534,780 ± 104,527	<0.001
		none	45,863 ± 563	992,097 ± 14,704	3,345,866 ± 112,215	<0.001
6	*Klebsiella pneumoniae* (4 μg/mL)	4	14,861 ± 729	18,396 ± 1,404	19,481 ± 798	0.001
		2	22,412 ± 150	35,850 ± 3,902	38,347 ± 1,229	0.001
		1	28,423 ± 611	35,553 ± 3,024	74,313 ± 1,108	<0.001
		0.5	34,349 ± 370	62,374 ± 2,283	1,188,054 ± 27,969	<0.001
		none	78,853 ± 1,441	1,535,944 ± 132,179	2,113,499 ± 89,930	<0.001
7	*Klebsiella oxytoca* (1 μg/mL)	4	3,111 ± 1,096	2,678 ± 371	1,811 ± 84	0.91
		2	2,808 ± 1,026	716 ± 104	384 ± 76	0.97
		1	3,237 ± 1,082	4,909 ± 408	1,418 ± 127	0.95
		0.5	3,256 ± 1,048	35,793 ± 2,711	152,479 ± 4,227	<0.001
		none	4,355 ± 760	78,130 ± 6,724	1,457,666 ± 4,494	<0.001
8	*Citrobacter freundii* (1 μg/mL)	4	4,274 ± 553	7,038 ± 174	5,176 ± 56	0.052
		2	4,245 ± 165	6,352 ± 132	2,711 ± 252	1.00
		1	4,698 ± 262	10,234 ± 612	4,140 ± 227	0.98
		0.5	5,036 ± 137	43,424 ± 824	527,459 ± 13,777	<0.001
		none	6,796 ± 190	318,993 ± 9,947	1,929,311 ± 70,098	<0.001

The unpaired upper one-sided Welch's t-test with a 0.05 significance level was used for the statistical analysis, with the ATP levels after 6-h incubation with levofloxacin (or the broth as a control) compared to those after 2-h incubation with the same concentration of LVFX. MIC = minimal inhibitory concentration, LVFX = levofloxacin, ATP = adenosine triphosphate.

Supplemental Table S3. The MIC values obtained by the comparison of 4-h time point and 2-h time point.

Cases #	Species (MIC values)	LVFX (μg/mL)	*p*-value	The MIC values at 4-h time point
1	*Escherichia coli* (≤0.5 μg/mL)	4	0.90	2.μg/mL
		2	0.91	
		1	0.013	
		none	0.057	
2	*Klebsiella pneumoniae* (≤0.5 μg/mL)	4	0.057	4 μg/mL
		2	0.001	
		1	0.034	
		none	0.002	
3	*Klebsiella pneumoniae* (1 μg/mL)	4	1.00	≤1 μg/mL
		2	1.00	
		1	0.93	
		none	0.009	
4	*Enterobacter cloacae* (≤0.5 μg/mL)	4	0.016	>4 μg/mL
		2	<0.001	
		1	0.005	
		none	0.002	
5	*Morganella morganii* (≤0.5 μg/mL)	4	0.97	4 μg/mL
		2	0.003	
		1	0.93	
		none	0.006	
6	*Salmonella enterica* (≤0.5 μg/mL)	4	1.00	<1 μg/mL
		2	0.99	
		1	0.93	
		none	0.014	
7	*Staphylococcus aureus* (≤0.5 μg/mL)	4	0.81	<1 μg/mL
		2	0.54	
		1	0.26	
		none	0.017	

Cases #	Species (MIC values)	LVFX (μg/mL)	*p*-value	The MIC values at 4-h time point
8	*Staphylococcus epidermidis* (4 μg/mL)	4	0.04	>4 μg/mL
		2	0.008	
		1	0.051	
		none	0.005	
9	*Staphylococcus epidermidis* (>4 μg/mL)	4	0.004	>4 μg/mL
		2	0.035	
		1	0.048	
		none	0.009	
10	*Staphylococcus capitis* (4 μg/mL)	4	0.004	>4 μg/mL
		2	0.047	
		1	0.004	
		none	0.02	
11	*Streptococcus pyogenes* (>8 μg/mL)	4	0.004	>4 μg/mL
		2	0.006	
		1	0.010	
		none	0.012	
12	*Streptococcus agalactiae* (0.5 μg/mL)	4	0.18	<1 μg/mL
		2	0.19	
		1	0.12	
		none	0.21	
13	*Streptococcus mitis* (1 μg/mL)	4	0.32	4 μg/mL
		2	0.007	
		1	0.041	
		none	0.004	
14	*Enterococcus faecium* (>4 μg/mL)	4	0.10	>4 μg/mL
		2	0.001	
		1	0.001	
		none	0.003	
15	*Enterococcus faecium* (>4 μg/mL)	4	0.036	>4 μg/mL
		2	0.006	
		1	0.0017	
		none	0.004	

The unpaired upper one-sided Welch's t-test with a 0.05 significance level was used for the statistical analysis, with the ATP levels after 4-h incubation with levofloxacin (or the broth as a control) compared to those after 2-h incubation with the same concentration of LVFX. MIC = minimal inhibitory concentration, LVFX = levofloxacin, ATP = adenosine triphosphate.
